# Suppression of nitric oxide induction and pro-inflammatory cytokines by novel proteasome inhibitors in various experimental models

**DOI:** 10.1186/1476-511X-10-177

**Published:** 2011-10-12

**Authors:** Asaf A Qureshi, Xiaoyu Tan, Julia C Reis, Mostafa Z Badr, Christopher J Papasian, David C Morrison, Nilofer Qureshi

**Affiliations:** 1 Department of Basic Medical Science, School of Medicine, University of Missouri-Kansas City, 2411 Holmes Street, Kansas City, MO 64108, USA; 2 Department of Medicine, University of Kansas Medical Center, 3901 Rainbow Boulevard, Kansas City, KS 66160, USA; 3 Division of Pharmacology and Toxicology, School of Pharmacy, University of Missouri-Kansas City, 2464 Charlotte Street, Kansas City, MO 64108, USA

## Abstract

**Background:**

Inflammation has been implicated in a variety of diseases associated with ageing, including cancer, cardiovascular, and neurologic diseases. We have recently established that the proteasome is a pivotal regulator of inflammation, which modulates the induction of inflammatory mediators such as TNF-α, IL-1, IL-6, and nitric oxide (NO) in response to a variety of stimuli. The present study was undertaken to identify non-toxic proteasome inhibitors with the expectation that these compounds could potentially suppress the production of inflammatory mediators in ageing humans, thereby decreasing the risk of developing ageing related diseases. We evaluated the capacity of various proteasome inhibitors to suppress TNF-α, NO and gene suppression of TNF-α, and iNOS mRNA, by LPS-stimulated macrophages from several sources. Further, we evaluated the mechanisms by which these agents suppress secretion of TNF-α, and NO production. Over the course of these studies, we measured the effects of various proteasome inhibitors on the RAW 264.7 cells, and peritoneal macrophages from four different strains of mice (C57BL/6, BALB/c, proteasome double subunits knockout LMP7/MECL-1^-/-^, and peroxisome proliferator-activated receptor-α,^-/- ^(PPAR-α,^-/-^) knockout mice. We also directly measured the effect of these proteasome inhibitors on proteolytic activity of 20S rabbit muscle proteasomes.

**Results:**

There was significant reduction of chymotrypsin-like activity of the 20S rabbit muscle proteasomes with dexamethasone (31%), mevinolin (19%), δ-tocotrienol (28%), riboflavin (34%), and quercetin (45%; ***P ***< 0.05). Moreover, quercetin, riboflavin, and δ-tocotrienol also inhibited chymotrypsin-like, trypsin-like and post-glutamase activities in RAW 264.7 whole cells. These compounds also inhibited LPS-stimulated NO production and TNF-α, secretion, blocked the degradation of P-IκB protein, and decreased activation of NF-κB, in RAW 264.7 cells. All proteasome inhibitors tested also significantly inhibited NO production (30% to 60% reduction) by LPS-induced thioglycolate-elicited peritoneal macrophages derived from all four strains of mice. All five compounds also suppressed LPS-induced TNF-α, secretion by macrophages from C57BL/6 and BALB/c mice. TNF-α, secretion, however, was not suppressed by any of the three proteasome inhibitors tested (δ-tocotrienol, riboflavin, and quercetin) with LPS-induced macrophages from LMP7/MECL-1^-/- ^and PPAR-α,^-/- ^knockout mice. Results of gene expression studies for TNF-α, and iNOS were generally consistent with results obtained for TNF-α, protein and NO production observed with four strains of mice.

**Conclusions:**

Results of the current study demonstrate that δ-tocotrienol, riboflavin, and quercetin inhibit NO production by LPS-stimulated macrophages of all four strains of mice, and TNF-α, secretion only by LPS-stimulated macrophages of C57BL/6 and BALB/c mice. The mechanism for this inhibition appears to be decreased proteolytic degradation of P-IκB protein by the inhibited proteasome, resulting in decreased translocation of activated NF-κB to the nucleus, and depressed transcription of gene expression of TNF-α, and iNOS. Further, these naturally-occurring proteasome inhibitors tested appear to be relatively potent inhibitors of multiple proteasome subunits in inflammatory proteasomes. Consequently, these agents could potentially suppress the production of inflammatory mediators in ageing humans, thereby decreasing the risk of developing a variety of ageing related diseases.

## Background

Modern industrialized societies are experiencing great increases in many age-related diseases such as diabetes, cardiovascular, neurodegenerative diseases, and certain types of cancer. Although numerous factors undoubtedly contribute to this trend, significant evidence implicates nitric oxide (NO), and inflammation, in the pathogenesis of several of these age-related diseases [1]. A number of studies, using experimental animal models, have demonstrated that senescence is accompanied by increases in production of NO in response to a variety of microbial products. For example, lipopolysaccharide (LPS)-induced macrophages from 22 and 32 month old CBA/CA mice to produce approximately 5 fold and 15 fold more NO, respectively, than LPS-stimulated macrophages from young (2-month-old) CBA/CA mice [2]. Through further exploration of innate inflammatory responses we have learned that the kinetics of NO production and TNF-α secretion differ in LPS-stimulated murine macrophages, that induction of these inflammatory products are regulated by two independent signaling pathways, and that cytoplasmic proteasomes are key regulators of LPS-induced inflammatory responses in macrophages [3-7].

We have recently reviewed the important role of proteasomes in inflammation and other macrophage functions, and hypothesized that inhibition of proteasome activity can suppress inflammatory responses that contribute to ageing [8]. Many of our earlier experiments designed to delineate the role of proteasomes in innate inflammatory responses utilized lactacystin, a potent proteasome inhibitor [7]. Lactacystin is a synthetic compound that contains a β-lactone moiety, which is responsible for lactacystin's capacity to block production of a number of pro-inflammatory cytokines by LPS-stimulated macrophages [7]. Unfortunately, lactacystin is very expensive and toxic even at micromolar levels so, although it has been quite useful for *in vitro *experimentation, it is not suitable for clinical use [7]. As reported recently, proteasomal activities are tightly regulated, and naturally-occurring compounds (γ-tocotrienol and δ-tocotrienol) are able to inhibit or activate these activities [9].

Consequently, we sought to identify other, non-toxic proteasome inhibitors with anti-inflammatory properties. Specifically, we have been evaluating a number of relatively inexpensive, commercially available naturally-occurring, synthetic, and FDA approved compounds for their capacity to inhibit proteasome activity, and the production of nitric oxide, certain pro-inflammatory cytokines (TNF-α, IL-1β, IL-6), and the iNOS enzyme. As part of this pursuit, we recently reported that two important inflammatory markers associated with ageing, TNF-α and NO, were effectively decreased in chickens whose diets were supplemented with a variety of naturally-occurring, synthetic or FDA approved compounds such as δ-tocotrienol, quercetin, riboflavin, (-) Corey lactone, amiloride, and dexamethasone [10]. As described earlier, NO production increases during ageing process [2], which could be due to a diminished activation of NF-κsignaling [11,12]. It was suggested that above mentioned compounds may also block the activation of NF-κ, thus resulting lowering of serum TNF-α and NO levels in chickens [10]. The important role played by NF-κin various biological functions has been described [13].

**Figure 1:**
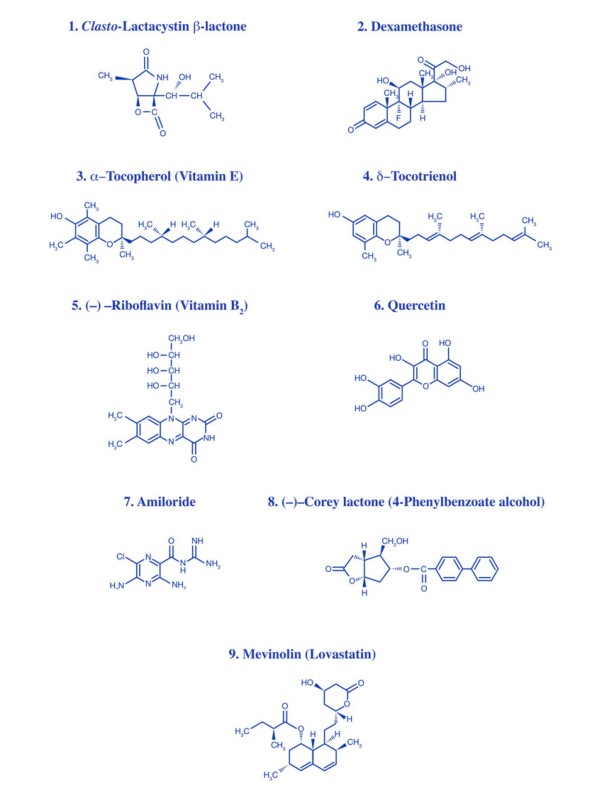
**Chemical structures of various compounds used in this study**.

The objective of the present study was to determine the possible mechanisms by which δ-tocotrienol, quercetin, riboflavin (vitamin B_2_), mevinolin, dexamethasone (as positive controls), and α -tocopherol (vitamin E) suppress LPS-induced inflammatory responses. Dexamethasone, mevinolin, δ-tocotrienol, α -tocopherol, quercetin, and riboflavin were selected due to the presence of a lactone moiety, similar to that of lactacystin, as shown in Figure 1. We were specifically interested in determining the extent to which these compounds impact proteasome activities dependent on 6 protease-active sites (i.e. X, Y, Z, LMP7, LMP2 and MECL-1^-/-^), and signaling pathways (i.e. inhibition of NF-κ, cleavage of P-Iκ) leading to LPS-induced production of NO and TNF-α secretion in murine macrophages. The present studies were conducted using various types of macrophages, including a macrophage like murine cell line (RAW 264.7), and thioglycolate-elicited peritoneal macrophages from C57BL/6 (normal), BALB/c, "proteasome double subunits knockout" (lacking LMP-7^-/-^/MECL-1^-/- ^subunits of the proteasomes), and peroxisome proliferator-activated receptor-α^-/- ^(PPAR-α^-/-^) deficient mice. PPAR-α^-/- ^deficient mice were investigated because they exhibit a prolonged response to inflammatory stimuli [14,15].

## Materials and methods

### Reagents

Highly purified, deep rough chemotype LPS (Re LPS) from *E. coli *D31m4 was prepared as described [4]. Dulbecco's Modified Eagle Medium (DMEM) heat-inactivated low-endotoxin fetal bovine serum (FBS), and gentamicin were purchased from Cambrex (Walkersville, MD, USA) for tissue culture studies. Thioglycollate was purchased from Sigma, Aldrich Chemical Co. (St. Louis, MO, USA) and RNeasy mini kit from QIAGEN Sciences (Germantown, MD, USA). RAW 264.7 cells (ATCC TIB 71) were purchased from American Type Culture Collection (Manassas, VA, USA). Mevinolin, pure α,-tocopherol, (-) Corey lactone, amiloride-HCL, (-) riboflavin and dexamethasone were purchased from Sigma-Aldrich Chemical Co (St. Louis, MO, USA). Quercetin was purchased from Alfa Aesar (Johnson Matthey Co. Lancastor, UK). The 50% purified δ-tocotrienol fraction from annatto seeds was received as a gift from American River (Boston, MA, USA).

### Purification of δ-tocotrienol from 50% purified fraction of annatto seeds

The δ-tocotrienol was purified from 50% purified fraction of annatto seeds, as described previously [16]. The purity of δ-tocotrienol was established by high pressure liquid chromatography (HPLC) against its standard, as reported earlier [16].

### Cell culture and maintenance

The RAW 264.7 cells or mouse thioglycolate-elicited peritoneal macrophages were maintained in DMEM supplemented with 10% heat inactivated FBS and 10 mg/mL gentamicin at 37°C in a humidified atmosphere with 5% CO_2_, as described previously [3]. Cells were cultured in 6-well plates as described in the Tables or legends to the Figures.

### Animals

The 6-week-old female, C57BL/6, Wild Type (WT) and BALB/c, were obtained from the Jackson Laboratory (Bar Harbor, ME, USA). 6-week-old female proteasome double subunits knockouts (LMP7/MECL-1^-/-^) mice were obtained from Dr. John J Monaco (Department of Molecular Genetics, University of Cincinnati Medical Center, Cincinnati, OH, USA) and peroxisome proliferator-activated receptor-α,^-/- ^(PPAR-α,^-/-^) were bred at UMKC's Animal Facility (Kansas City, MO, USA).

Mice used in this study received humane care in compliance with the principles of laboratory animal care formulated by the National Society of Health Guide for the "Care and Use of Laboratory Animals" by the US National Society of Health (NIH Publication No 85-23, revised 1996).

The experimental procedures involving animals were reviewed and approved by the "Institutional Animal Care and Use Committee of UMKC", Medical School, MO. All 6-week-old female mice (*n *= 20) were acclimatized to the new environment for 14 days before beginning experimentation. The mice were fed ad libitum regular commercial mouse diet and had free access to water throughout the experiment. A 12 h light and 12 h dark cycle was maintained during feeding period.

### Chymotrypsin-like activity of 20S rabbit muscle proteasomes

Comparative effectiveness of different doses (5 μM-320 μM) of dexamethasone, mevinolin, α,-tocopherol, δ-tocotrienol, riboflavin, quercetin-HCL, amiloride-HCL, and (-) Corey lactone on the suppression of chymotrypsin-like activity of 20S rabbit muscle proteasomes was evaluated. For these studies, different concentrations of each compound were prepared in media containing 0.5% DMSO. The proteasomal activities of the 20S rabbit muscle proteasomes (0.4 μg/mL) were assayed with synthetic peptide substrate in 0.02 M Tris-HCl buffer (pH 7.2). The substrate used for the chymotrypsin-like activity was 100 μM of succinyl-Leu-Leu-Val-Tyr-amino methyl coumarin. Fluorescence was measured (absorption at 360 nm and emission at 460 nm) using an Flx 800 microplate fluorescence reader (Bio-Tek Instruments, Winooski, VT, USA).

### Effects of quercetin, riboflavin, and δ-tocotrienol (concentrations of 5 μM-40 μM) after 60 min treatment of different proteasomal activities (chymotrypsin-like, trypsin-like, post-glutamase) in RAW 264.7 whole cells

The comparative inhibitory effect of quercetin, riboflavin, and δ-tocotrienol on the chymotrypsin-like, trypsin-like, and post-glutamase activities of proteasome using RAW 264.7 whole cells were carried out essentially as reported recently [9]. Briefly RAW 264.7 cells (10 × 10^3 ^cells/100 μL/well) were added in white plates (96-well, Fisher, 0877126), followed by the addition of various concentrations of quercetin, riboflavin, or δ-tocotrienol (5, 10, 20, or 40 μM in 100 μL; dissolved in 0.4% dimethyl sulfoxide (DMSO). The mixtures were incubated at 37°C in an incubator at 5% CO_2 _for 60 min. After incubation period, the cells in the 96-well plates were taken out 20 min prior to the addition of Caspase-Glo reagent (brought to room temperature before addition to the wells). Caspase-Glo reagent (100 μL) was added to each well to a total volume of 200 μL/well (tris buffer, pH 7.5; 0.02 M). The plate were covered with a plate sealer and incubated at room temp for 30 min. The relative luminescence units (RLU) of assays were read in a Promega Plate Luminometer. The chymotrypsin-like, trypsin-like, or post-glutamase activities were quantitated by measuring luminescence after stimulation of RAW 264.7 whole cells with various concentration of each compound in a Luminometer (Promega, Madison, Wisconsin USA), according to the directions of manufacturer.

### TNF-α, secretion and NO production by LPS-induced RAW 264.7 cells and LPS-induced thioglycolate-elicited peritoneal macrophages of four strains of mice

#### LPS-induced RAW 264.7 cells

RAW 264.7 cells (1 × 10^6 ^cells/500 μL/well) were adhered for 2 h in the wells. After 2 h cells were treated with dexamethasone, mevinolin (positive controls), α,-tocopherol, δ-tocotrienol, riboflavin, or quercetin (100 μL; dissolved in 0.5% DMSO) for 1 h (pre-treatment). One hour later all wells were challenged with LPS (10 *ng*/well; 400 μL) or medium and incubated at 37°C in 5% CO_2 _for 4 h (TNF-α,) or 36 h (NO). After incubation, the supernatants were collected and stored at -20°C.

The levels of TNF-a in supernatants were measured by Quantikine M ELISA kit (R&D System, Minneapolis, MN, USA) according to manufacturer's instructions. The lower limit of detection for TNF-a in this method is approximately, 5.0 *pg*/mL [4,7].

The levels of NO were determined by measuring the amount of nitrite, a stable metabolic product of nitric oxide, as previously reported [17]. The assay mixture contained medium (100 μL) plus Griess reagent (100 μL), and absorption was measured at 570 nm using a "Microplate Reader" (MR 5000; Dynatech Labs, Inc. USA). The amount of nitrite was determined by comparison of unknowns using a NaNO2 standard curve. The NO detection limit was 0.20 nM [17].

#### LPS-induced thioglycolate-elicited peritoneal macrophages

The procedures described above are identical to those utilized for peritoneal macrophages except that thioglycolate-elicited peritoneal macrophages were adhered to the bottom of 100 mm tissue culture plates (1 × 10^7 ^cells/well in 1.0 mL media) for 4 h, the supernatants were removed, and cells were washed extensively with medium three times. The cells were cultured overnight in fresh media after the final wash. After overnight incubation at 37°C, the cells were treated with various proteasome inhibitors and LPS as described above. Viability of peritoneal macrophages treated with various inhibitors plus LPS were also determined by trypan blue dye exclusion or a quantitative colorimetric assay with 3-(4,5)-dimethylthiozol-2,5-diphenyl-tetrazolium bromide (MTT) as described previously [4,7].

### Effects of various compounds on the inhibition of NF-κB in LPS-stimulated RAW 264.7 cells

RAW 264.7 cells were pretreated with dexamethasone, mevinolin, α -tocopherol, δ-tocotrienol, riboflavin, or quercetin for 1 h, then treated with LPS (10 *ng*/well; 400 μL) for 4 h. Nuclear protein was extracted and the NF-κactivation was measured using "electronic mobility shift assay" (EMSA) according to the reported procedure [18]. Nuclear protein was extracted following the recommendation of the manufacturer (QIAGEN Sciences; Germantown, MD, USA) [19]. NF-κB was measured in nuclear extracts with the respective ELISA-based commercial kits (NF-κp65). Nuclear protein (5 μg), was added to each well coated with an oligonucleotide containing the consensus binding site for NF-κincubated for I h. Activated NF-κwas detected after 1 h with primary antibody, i.e. Anti-NF-κ, which specifically recognizes an epitope (p65) accessible only when the factor is activated and bound to its target DNA. A secondary anti-IgG horseradish peroxidase conjugate allows detection of the activated NF-κby a colorimetric reaction. Absorption was read within 5 min at 450 nm with a reference wavelength of 655 nm [19].

### Degradation of P-IκB, protein in LPS-stimulated RAW 264.7 cells (Western blots analyses)

RAW 264.7 cells (1 × 10^6 ^cells/500 μL/well) were treated with dexamethasone, mevinolin, α,-tocopherol, δ-tocotrienol, riboflavin, or quercetin for 1 h, then stimulated with LPS (10 *ng*/well; 400 μL) for 36 h. Macrophages were washed with phosphate-buffered saline, and cytoplasmic extracts were prepared using cell extraction buffer (Biosource, Camarillo, California, USA) supplemented with a protease inhibitor cocktail, containing phenylmethylsulfonyl fluoride and phosphatase inhibitors, according to the manufacturer's directions [20]. Protein concentrations were measured with BCA protein assay kits, and Western blots analyses were used to measure IκB-α, (Santa Cruz). Each well of the gel was loaded with 40 μg of protein and gels were electrophoresed at a constant 150 V 1 × Tris glycine buffer for 50 minutes. Proteins in gels were transferred into the "Immobilon Transfer Membranes" (IPVH 15150; Millipore, Bedford, Mass, USA) using the semidry transfer cell and, after appropriate antibody treatments, the bands were visualized with an enhanced chemiluminescence detection kit (Pierce) as described previously [7,20].

### Expression of TNF-α, and iNOS genes (Southern blots analyses)

All proteasome inhibitors (α,-tocopherol, δ-tocotrienol, riboflavin, or quercetin) were dissolved in media containing 0.2% DMSO. Thioglycolate-elicited peritoneal macrophages were prepared from 8-week-old mice as described previously (7,17). The macrophages (1 × 10^7^) were adhered for 2 h in the wells, with α,-tocopherol (100 μM), δ-tocotrienol (10 μM), riboflavin (40 μM), or quercetin (40 μM) for 2 h. Then all the wells were challenged with LPS (10 *ng*/well; 400 μL), and incubated at room temperature for 4 h.

After 4 h, assay mixtures were centrifuged at 2,000 rpm for 20 min. Cells were harvested, and total cellular RNA was extracted from each pellet with RNeasy mini kit (QIAGEN Sciences; Germantown, MD, USA) according to the instructions of the manufacturer. The RNA of each treatment was transcribed and resulting data was amplified and analyzed by real-time polymerase chain reaction (RT-PCR) to quantitate gene expression of TNF-α, and iNOS by using 1-step RT-PCR kit (QIAGEN, Chatsworth, CA, USA; Southern blots analyses) according to the manufacturer's instructions [6,7,21].

### Detection of cell viability

Viability of peritoneal macrophages treated with and without LPS plus dexamethasone, mevinolin, α,-tocopherol, δ-tocotrienol, riboflavin, and quercetin-HCL was determined by trypan blue dye exclusion or a quantitative colorimetric assay with 3-(4,5)-dimethyl-thiozol-2, 5-diphenyltetrazolium bromide (MTT) as reported [4,7].

### Statistical Analyses

Stat View software (version 4.01, Abacus Concepts, Berkeley, CA) was used for the analyses of treatment-mediated effects as compared to control group. Treatment-mediated differences were detected with a two-way ANOVA, and when the F test indicated a significant effect, differences between the means were analyzed by a Fisher's protected least significant difference test. Data were reported as means ± SD in text and Tables. The statistical significance level was set at 5% (***P ***< 0.05).

## Results

### Effects of various compounds on the chymotrypsin activity of 20S rabbit muscle proteasomes

The anti-inflammatory effects of δ-tocotrienol, quercetin, riboflavin, (-) Corey lactone, amiloride and dexamethasone, as determined by effects on serum levels of TNF-α, and nitric oxide in chickens, have been reported recently [10]. In order to determine whether these compounds modulate proteasome activity, chymotrypsin-like activity of 20S rabbit muscle proteasomes was measured after treatment with these compounds at concentrations ranging from 5 μM to 320 μM, and compared to activity of vehicle treated controls. Results of this study revealed dose dependent inhibition of chymotrypsin-like activity of 20S rabbit muscle proteasomes between 5 μM and 40 μM for mevinolin and δ-tocotrienol, and between 5 μM to 320 μM for dexamethasone, quercetin, and riboflavin (Table 1). The inhibitory effects of mevinolin and δ-tocotrienol were reversed at higher concentrations between 80 μM and 320 μM (Table 1). (-) Corey lactone and amiloride-HCL induced dose-dependent (5 μM to 320 μM) increases of chymotrypsin-like activity of 20S rabbit muscle proteasomes, whereas α,-tocopherol (vitamin E) had no effect on at the concentrations tested (Table 1).

**Table 1:** Effects of various compounds on the chymotrypsin-like activity of 20S rabbit muscle proteasomes^1^

	Treatment				Absorption^2^				
**#**	**Assay Mixture**	**Dexame**	**Mevino**	**α,-Tocop**	**δ-Tocotri**	**Ribofl**	**Querce**	**Amilor**	**(-) Cor Lact**

1	20S Prot (20S P)	28486	28486	28486	28486	28486	28486	28486	28486
2	Tris + 20S P = A	25259	25259	25259	25259	25259	25259	25259	25259
3	A + DMSO^3 ^= B	27792 (100)^4^	27792 (100)^4^	27792 (100)^4^	27792 (100)^4^	27792 (100)^4^	27792 (100)^4^	27792 (100)^4^	27792 (100)^4^
4	B + 5 μM	22340 (81)	24120 (87)	27389 (99)	21348 (77)	24567 (88)	21670 (78)	33072 (119)	31961 (115)
5	B + 10 μM	19250 (69)	2439 (88)	26890 (97)	20122 (72)	22459 (81)	18768 (68)	55306 (199)	34740 (125)
6	B + 20 μM	16789 (60)	22412 (81)	27560 (99)	15904 (57)	20670 (74)	16342 (59)	77262 (278)	38353 (138)
7	B + 40 μM	15490 (56)	18704 (67)	27180 (98)	16458 (59)	18272 (66)	15246 (55)	115336 (415)	44745 (161)
8	B + 80 μM	12540 (45)	19870 (71)	27141 (98)	18690 (67)	17650 (64)	12560 (45)	178147 (641)	39187 (141)
9	B + 160 μM	9635 (35)	23460 (84)	26870 (97)	21405 (77)	15890 (57)	11345 (41)	191209 (688)	51415 (185)
10	B + 320 μM	8726 (31)	23960 (86)	26560 (96)	25670 (92)	12780 (46)	9650 (35)	219557 (790)	65033 (234)

Dexame = Dexamethasone; Mevino = Mevinolin; α,-Tocop = α,-Tocopherol; δ-Tocotri = δ-Tocotrienol; Ribofl = Riboflavin; Querce = Quercetin;

Amilor = Amiloride; (-) Cor Lact = (-) Corey Lactone; 20S Prot = 20S Proteasomes.

^1^Chymotrypsin-like activity was carried out by using synthetic dipeptide substrate III (Suc-Leu-Leu-Val-Tyr-AMC) in Tris buffer pH 7.5, 0.02 M. 20S rabbit muscle proteasomes was used. Time of incubation was 30 minutes.

^2^Fluorescence absorption was measured at excitation = 360 nm and emission = 460 nm.

^3^Each compound was dissolved in dimethyl sulfoxide (0.5% DMSO). Appropriate amount of each compound (highest concentration) was dissolved in 1.0 mL DMSO = A stock solution; 50 μL of stock solution A was mixed with 950 μL of media = B solution. The remaining concentrations were prepared from B solution (1:2 dilution) successively.

^4^Percentage of control values are in parentheses.

**Figure 2:**
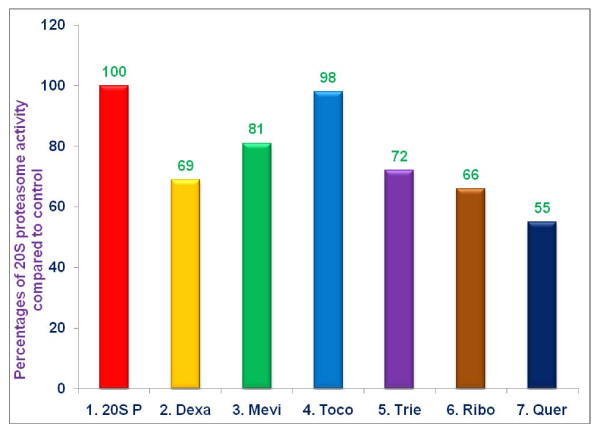
**Effects of proteasome inhibitors on chymotrypsin-like activity of 20S rabbit muscle proteasomes**: 20S Rabbit muscle proteasomes were treated with various compounds with 100 μL dissolved in 0.5% DMSO of dexamethasone (10 μM); mevinolin (20 μM); α,-tocopherol (100 μM); δ-tocotrienol (10 μM); riboflavin (40 μM); or quercetin-HCL (40 μM) for 30 min. Then, proteolytic activity was measured by adding succinyl-Leu-Leul-Val-Tyr-amino methyl coumarin as substrate and measuring fluorescence (absorption at 360 nm and emission at 460 nm) using an Flx 800 microplate fluorescence reader. The treatments 1-7 correspond to: 1. Control (tris + 20S proteasomes + 0.5% DMSO); 2. dexamethasone; 3. mevinolin; 4. α,-tocopherol; 5. δ-tocotrienol; 6. riboflavin; 7. quercetin-HCL.

The results presented in Table 1 demonstrate the capacity of several of these compounds to inhibit 20S chymotrypsin-like activity of rabbit muscle proteasomes at various concentrations. For subsequent experiments, we limited testing to a single concentration for each of these compounds. The capacity of these compounds to inhibit 20S chymotrypsin-like activity of rabbit muscle proteasomes at concentrations selected for subsequent experimentation is presented in Figure 2. Confirming our earlier results that dexamethasone (10 μM), mevinolin (20 μM), δ-tocotrienol (10 μM), riboflavin (40 μM), and quercetin (40 μM), all significantly inhibited proteasome activity (***P ***< 0.05), while α,-tocopherol (100 μM) did not have an effect [10]. The extent of proteasome inhibition, compared to controls, obtained with dexamethasone, mevinolin, δ-tocotrienol, riboflavin, and quercetin was 31%, 19%, 28%, 34%, and 45%, (***P ***< 0.05), respectively (Figure 2).

According to recent reports, the 20S proteasomes contain three distinct subunits (X, Y, and Z), with well characterized protease sites; subunits × (β5), Y (β1), and Z (β2) display chymotrypsin-like, trypsin-like, and post-glutamase actitivities, respectively [22]. In order to confirm that these naturally-occurring compounds (quercetin, riboflavin, and δ-tocotrienol) act as proteasome inhibitors, we measured their effects on chymotrypsin-like, trypsin-like and post-glutamase proteasome activities. These enzymatic activities in RAW 264.7 cells were inhibited in a dose-dependent manner at concentrations between 5 μM and 40 μM (Table 2). Quercetin and δ-tocotrienol caused 50% inhibition of chymotrypsin-like activity with 40 μM concentration compared to control, whereas, riboflavin resulted only moderate inhibition of 14% with this dose (Table 2). Quercetin was the most potent inhibitor of trypsin-like (62%) and post-glutamase (41%) activities compared to riboflavin and δ-tocotrienol treatments (Table 2), thus confirming our earlier results that these compounds modulate inhibition as proteasome inhibitors [10].

**Table 2:** Effect of quercetin, riboflavin, and δ-tocotrienol (concentrations of 5 μM-40 μM) in RAW 264.7 cells on proteasomal activities (chymotrypsin, trypsin, post-glutamase) for a duration of 1 h^1^

#	Assay mixture^2^	Chmotrypsin-like	Ttypsin-like	Post-glutamase
		**Avg RLU value**	**Avg RLU value**	**Avg RLU value**

1	1. Media and Cells	107,890	92,168	101,874
2	2. DMSO Control^3^	139,693 (100)^4^	107,753 (100)^4^	106,034 (100)^4^
				
	**Quercetin-HCL**			
				
3	5 μM	133,414 (96)	84,039 (78)	105,301 (99)
4	10 μM	133,089 (95)	80,517 (75)	102,821 (97)
5	20 μM	116,890 (84)	69,853 (65)	99,211 (94)
6	40 μM	70,543 (50)	40,776 (38)	63,012 (59)
				
	**Riboflavin**			
				
7	5 μM	143,069 (102)	88,251 (82)	102,858 (96)
8	10 μM	131,181 (94)	86,255 (80)	101,169 (95)
9	20 μM	120.900 (87)	83,533 (78)	100,199 (94)
10	40 μM	120,720 (86)	80,054 (74)	97,495 (92)
				
	δ-Tocotrienol*			
				
11	5 μM	119,147 (85)	93,331 (87)	106,844 (101)
12	10 μM	116,001 (83)	91,939 (85)	103,108 (97)
13	20 μM	114,996 (82)	86,700 (80)	95,236 (90)
14	40 μM	77,882 (56)	86,006 (80)	88,735 (84)

^1^Proteasome-Glo (Promega) chymotrypsin-Like, trypsin-Like, and post-glutamase cell-based assays were used. 10,000 cells were plated per well (in 100 μL of media) in a 96-well white plate. Cells were allowed to adhere to plates for 2 h prior testing.

^2^Each assay were carried out according to Promega Protocols, and plates were read with a Luminometer to give relative luminescence units (RLU) values.

^3^Media + cells + 0.4% dimethyl sulfoxide (DMSO) was used as control.

All concentrations of each compound contain 0.4% DMSO. ^4^Percentages of control values are in parenthesis.

*Similar values were observed for this range of reference [9] in *Lipids in Health and Disease *2010, **9:**143.

### Effects of various proteasome inhibitors on production of TNF-α, and NO by LPS-stimulated RAW 264.7 cells

**Figure 3:**
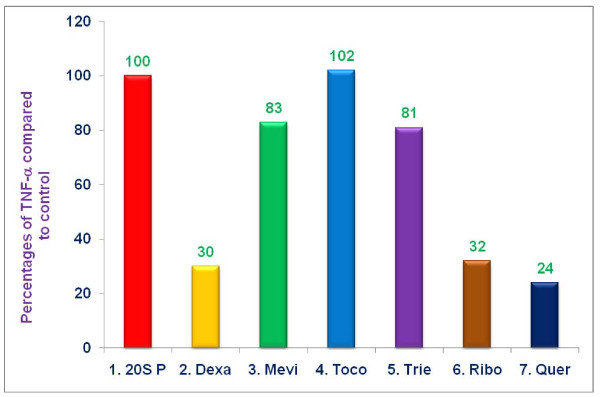
**The effect of proteasome inhibitors on TNF-α, secretion by LPS-stimulated RAW 264.7 cells**. RAW 264.7 cells (1 × 10^6 ^cells/500 μL/well) were adhered for 2 h in the wells, after 2 h, the cells were treated with 100 μL dissolved in 0.5% DMSO of dexamethasone (10 μM), mevinolin (20 μM), α,-tocopherol (100 μM), δ-tocotrienol (10 μM), riboflavin (40 μM), or quercetin-HCL (40 μM) for 1 h. Then all wells were challenged with LPS (10 *ng*/well; 400 μL) and incubated at 37°C 5% CO_2 _for 4 h. Supernatants were assayed for TNF-α, secretion by ELISA kit. Data are presented as the percent of TNF-α, levels compared to controls. The kit control TNF-α, value was 287. The treatments 1-7 correspond to: 1. Control (media + cells + LPS [10 *ng*/mL] + 0.5% DMSO); 2. dexamethasone; 3. mevinolin; 4. α,-tocopherol; 5. δ-tocotrienol; 6. riboflavin; 7. quercetin-HCL.

The anti-inflammatory properties of dexamethasone (10 μM), mevinolin (20 μM), δ-tocotrienol (10 μM), riboflavin (40 μM), quercetin (40 μM), and α,-tocopherol (100 μM) were explored by pre-treating RAW 264.7 macrophage-like cells for 1 h with each of these compounds followed by stimulation with LPS and measurement of TNF-α, or nitrite in culture supernatants 4 h and 36 h, respectively, after LPS stimulation. Quercetin (76%), dexamethasone (70%), and riboflavin (68%) significantly inhibited LPS-induced secretion of TNF-α, whereas, δ-tocotrienol and mevinolin produced only moderate reductions of 19% and 17%, respectively as compared to controls (Figure 3). Our previous studies with LPS-stimulated macrophages demonstrated that lactacystin, which inhibits primarily chymotrypsin-like activity of the proteasome, did not significantly inhibit TNF-α, production (22). In order to suppress TNF-α, secretion by LPS- stimulated macrophages, both the chymotrypsin-like and trypsin-like enzymatic proteasomal activity of the proteasome had to be inhibited (22). Thus, in the current experiments (Figure 3), the capacity of the proteasome inhibitors to inhibit TNF-α, secretion by LPS-stimulated macrophages further supports the conclusion that these inhibitors suppress both chymotrypsin-like and trypsin-like activities of the proteasome.

We also tested the ability of proteasome inhibitors to suppress TNF-α, secretion by LPS-stimulated macrophages under conditions in which macrophages were treated simultaneously with proteasome inhibitors and LPS. We found that the degree of inhibition of TNF-α, secretion by simultaneous treatment with LPS and quercetin (74%), dexamethasone (66%), riboflavin (65%), δ-tocotrienol (16%), or mevinolin (11%) (data not presented) was comparable to the level of inhibition attained when macrophages were pre-treated with proteasome inhibitors. Consequently, further studies were carried out only under pre-treatment condition.

**Figure 4:**
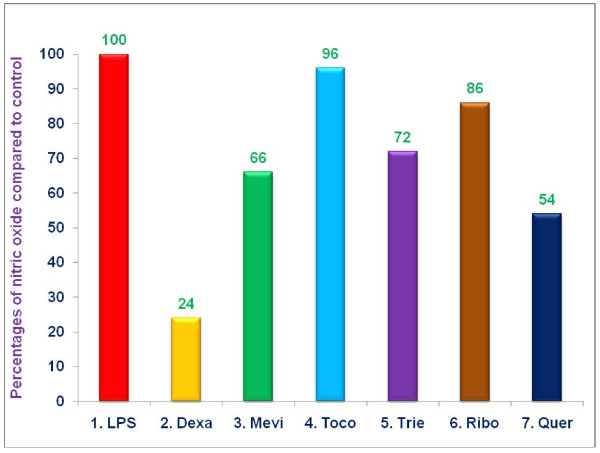
**The effect of proteasome inhibors on NO production by LPS-stimulated RAW 264.7 cells**. RAW 264.7 cells (1 × 10^6 ^cells/500 μL/well) were adhered for 2 h in the wells, after 2 h, the cells were treated with 100 μL dissolved in 0.5% DMSO of dexamethasone (10 μM), mevinolin (20 μM), α,-tocopherol (100 μM), δ-tocotrienol (10 μM), riboflavin (40 μM), or quercetin-HCL (40 μM) for 1 h. Then all wells were challenged with LPS (10 *ng*/well; 400 μL) and incubated at 37°C 5% CO_2 _for 36 h. Supernatants were assayed for the production of NO by measuring the amount of nitrite using the Griess reagent. Data are presented as the percent of NO levels compared to controls. The treatments 1-7 correspond to: 1. Control (media + cells + LPS [10 *ng*/mL] + 0.5% DMSO); 2. dexamethasone; 3. mevinolin; 4. α,-tocopherol; 5. δ-tocotrienol; 6. riboflavin; 7. quercetin-HCL.

When testing the ability of proteasome inhibitors to block production of NO by LPS-stimulated RAW 264.7 cells, we found that dexamethasone (76%) and quercetin (46%) were the most efficient inhibitors, while mevinolin (34%), δ-tocotrienol (28%), and riboflavin (14%) blocked the production of NO only modestly (Figure 4). α,-Tocopherol had no significant effect on the levels of nitric oxide (Figure 4).

### Effects of various proteasome inhibitors on the inhibition of NF-κB in LPS-stimulated RAW 264.7 cells

**Figure 5:**
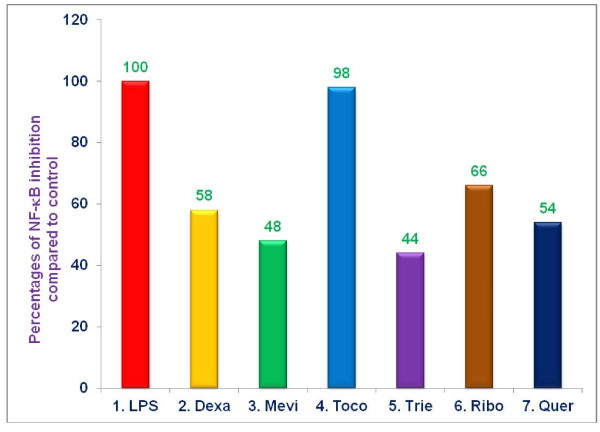
**The effect of proteasome inhibitors on translocation of NF-κB to the nucleus in LPS-stimulated RAW 264.7 cells**. RAW 264.7 cells (1 × 10^6 ^cells/500 μL/well) for 2 h, after 2 h, the cells were treated with 100 μL dissolved in 0.5% DMSO of dexamethasone (10 μM), mevinolin (20 μM), α,-tocopherol (100 μM), δ-tocotrienol (10 μM), riboflavin (40 μM), or quercetin-HCL (40 μM) for 1 h. Then all the wells were challenged with LPS (10 *ng*/well; 400 μL) and incubated at 37°C 5% CO_2 _for 36 h. At that time nuclear protein was extracted NF-κB activation was measured using "electronic mobility shift assay" (EMSA). Data are presented as the percent of nuclear NF-κB levels compared to controls. The treatments 1-7 correspond to: 1. Control (media + cells + LPS [10 *ng*/mL] + 0.5% DMSO); 2. dexamethasone; 3. mevinolin; 4. α,-tocopherol; 5. δ-tocotrienol; 6. riboflavin; 7. quercetin-HCL.

As described earlier, serum NO levels increase during ageing, and one potential explanation involves diminished regulation of NF-κB signaling with age [11,12]. Cytoplasmic NF-κB is usually bound to its inhibitor, IκB, and maintained in an inactive state. NF-κB becomes activated when IκB is phosphorylated, ubiquitinated, and degraded by the proteasome. NF-κB then migrates to the nucleus and binds to promoter sites of a variety of pro-inflammatory genes including TNF-α, and iNOS [13]. We hypothesized that increased NO levels, associated with ageing, may be attributable to increased degradation of IκB by ageing proteasomes, with resultant NF-κB activation. Consequently, we tested the ability of various proteasome inhibitors (dexamethasone, mevinolin, δ-tocotrienol, riboflavin, and quercetin) to inhibit activation of NF-κB, specifically using EMSA assays to detect NF-κB in nuclear extracts of LPS stimulated RAW-264.7 cells. Significant inhibition (***P ***< 0.02) of LPS-induced NF-κB activation, compared to medium controls, was achieved with δ-tocotrienol (56%), mevinolin (52%), quercetin (46%), dexamethasone (42%), and riboflavin (34%), while α,-tocopherol (vitamin E) had no effect (Figure 5). Thus, the capacity of each of these proteasome inhibitors to suppress LPS-induced NF-κB activation could account for their ability to inhibit TNF-α, secretion, and NO production.

### Effects of various proteasome inhibitors on levels of P-IκB in LPS-stimulated RAW 264.7 cells

**Figure 6:**
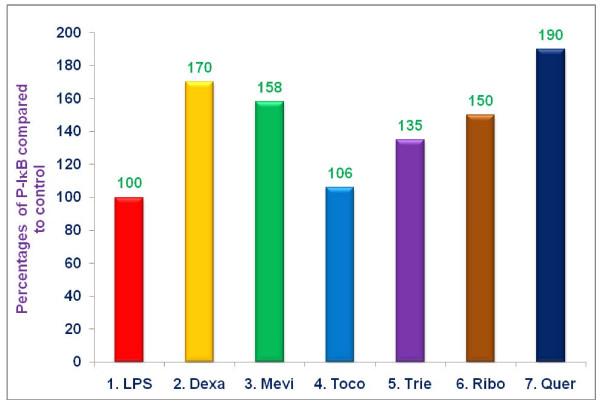
**The effect of proteasome inhibitors on intracellular levels of P-IκB, protein in LPS-stimulated RAW 264.7 cells (Western blots analyses)**. RAW 264.7 cells (1 × 10^6 ^cells/500 μL/well) for 2 h, after 2 h, the cells were treated with 100 μL dissolved in 0.5% DMSO of dexamethasone (10 μM), mevinolin (20 μM), α,-tocopherol (100 μM), δ-tocotrienol (10 μM), riboflavin (40 μM), or quercetin-HCL (40 μM) for 1 h. Then all the wells were challenged with LPS (10 *ng*/well; 400 μL) and incubated at 37°C 5% CO_2 _for 36 h. Cells were washed with phosphate buffer saline, and cytoplasmic extracts were prepared using cell extraction buffer supplemented with phenylmethylsulfonyl fluoride and phosphatase inhibitors, according to the directions of manufacture [20]. Protein concentrations were measured with BCA protein assay kits, and P-IκB-α, levels were measured by Western blots analyses [7,20]. Data are presented as the percent of P-IκB compared to controls. The treatments 1-7 correspond to: 1. Control (media + cells + LPS [10 *ng*/mL] + 0.5% DMSO); 2. dexamethasone; 3. mevinolin; 4. α,-tocopherol; 5. δ-tocotrienol; 6. riboflavin; 7. quercetin-HCL.

As mentioned above, phosphorylation of IκB is a prerequisite to its degradation by the proteasome, with resultant liberation, and activation of NF-κB. If the mechanism by which various proteasome inhibitors suppress activation of NF-κB in LPS-stimulated RAW 264.7 cells involves a decreased capacity to degrade P-IκB, levels of P-IκB would be expected to increase in LPS-stimulated RAW-264.7 cells treated with proteasome inhibitors, compared to vehicle treated controls. Pre-treatment of LPS-stimulated RAW 264.7 cells with quercetin, dexamethasone, mevinolin, riboflavin, and δ-tocotrienol resulted in significant increases of 90%, 70%, 58%, 50%, and 35% (***P ***< 0.05), respectively, compared to vehicle treated controls (Figure 6). Thus, the capacity of these proteasome inhibitors to suppress degradation of P-IκB probably accounts for their ability to inhibit activation of NF-κB, and secretion of TNF-α, and production of NO in LPS-stimulated RAW 264.7 cells.

### Effects of various proteasome inhibitors on the secretion of TNF-α, by LPS-stimulated peritoneal macrophages from BALB/c mice

In order to confirm the results obtained above using cell cultures, we determined the effects of these proteasome inhibitors on LPS-induced responses of thioglycolate-elicited peritoneal macrophages from several strains of mice. Serial dilutions (5 μM to 640 μM) of dexamethasone, mevinolin, α,-tocopherol, δ-tocotrienol, riboflavin, and quercetin, were tested for their ability to inhibit TNF-α, secretion by thioglycolate-elicited peritoneal macrophages of 8-week-old female BALB/c mice stimulated with LPS (Table 3). Comparable to results obtained with RAW 264.7 cells, significant inhibition of TNF-α, secretion was attained with all agents tested except α,-tocopherol. Based on these results, all further experiments were carried out using the following concentrations of these agents: dexamethasone (10 μM); mevinolin (20 μM); δ-tocotrienol (10 μM); riboflavin (40 μM), and; quercetin (40 μM).

**Table 3:** Effects of various compounds on the secretion of TNF-α, (*pg*/mL) in LPS-stimulated (pre-treatment) thioglycolate-elicited peritoneal macrophages of 8-week-old BALB/c female mice^1^

	Pre-treatment			Secretion of TNF-α, (*pg*/mL)			
**#**	**Assay Mixture**	**Dexamethasone**	**Mevinolin**	**α,-Tocopherol**	δ-Tocotrienol*	**Riboflavin**	**Quercetin**

1	Media + Cells = A						
2	A + LPS (10 ng/mL) = B	1224	1224	1224	1224	1224	1224
3	B + 0.2% DMSO^2 ^= C	1305 (100)^3^	1305 (100)^3^	1305 (100)^3^	1305 (100)^3^	1305 (100)^3^	1305 (100)^3^
4	C + 5 μM	333 (26)	1125 (86)	1280 (98)	938 (72)	1012 (76)	980 (75)
5	C + 10 μM	283 (22)	1064 (82)	1267 (97)	962 (74)	896 (69)	873 (67)
6	C + 20 μM	234 (18)	1012 (78)	1235 (95)	834 (64)	674 (52)	846 (65)
7	C + 40 μM	213 (16)	1048 (80)	1259 (96)	769 (59)	621 (48)	821 (63)
8	C + 80 μM	207 (16)	1140 (87)	1298 (99)	1673 (128)	544 (42)	778 (60)
9	C + 160 μM	133 (10)	1396 (107)	1323 (101)	1732 (133)	520 (40)	472 (36)
10	C + 320 μM	112 (9)	1443 (111)	1257 (96)	1987 (152)	480 (37)	345 (26)
11	C + 640 μM	85 (7)	1487 (114)	1241 (95)	2245 (172)	406 (31)	310 (24)

^1^The thioglycolate-elicited peritoneal macrophages (1.0 mL) were adhered to the bottom of 12 well plates (1 × 10^7 ^cells/well in 1.0 mL media) for 4 h, then cells were washed extennsively with medium 3-times. Then cells were cultured overnight in fresh media. After overnight incubation at 37°C in an incubator at 5% CO2 for 1 h, then cells were treated with different concentrations of various compounds for 1 h. after 1 h, LPS (10 μL of 1.0 μg/mL stock solution) was added to each well (LPS final concentration 10.0 *ng*/mL). The supernatants were collected after 4 h of incubation at room temperature. The supernatants were collected after 4 h of LPS challenge, and stored at -70°C to carry out TNF- α, ELISA assay. Cells viability were > 95% in all treatments.

^2^Each compound was dissolved in dimethyl sulfoxide (0.2% DMSO). Appropriate amount of each compound (highest concentration) was dissolved in 1.0 mL DMSO = A stock solution; 50 μL of stock solution A was mixed with 950 μL of media = B working solution. The remaining concentrations were prepared from B working solution (1:2 dilution) successively.

^3^Percentages of control values are in parenthesis.

*Values from reference [10] in *Lipids in Health and Disease *2011,**10:**39.

### Effects of various proteasome inhibitors on secretion of TNF-α, secretion and NO production by LPS-stimulated peritoneal macrophages from C57BL/6 versus BALB/c mice

**Figure 7:**
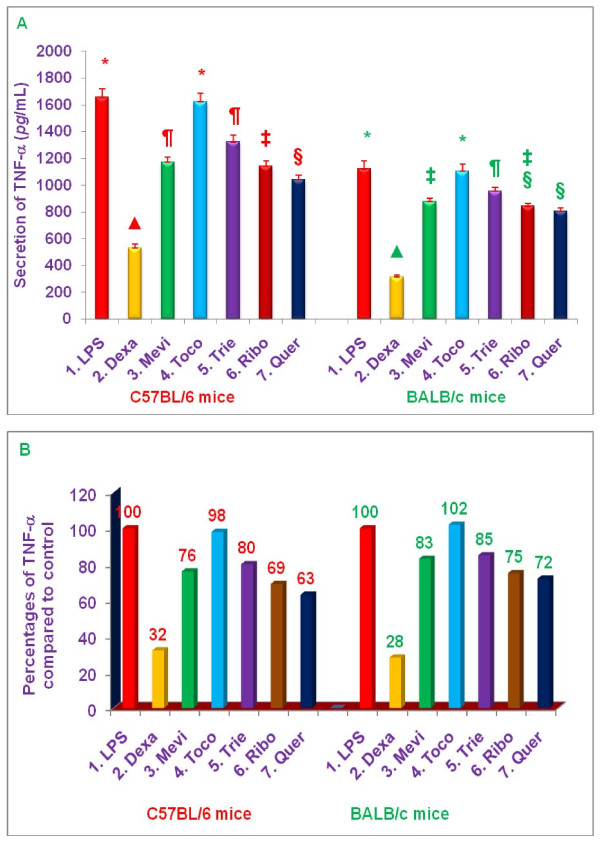
**Effects of various compounds on the secretion of TNF-α, in LPS-stimulated (pre-treatment) peritoneal macrophages of 8-week-old C57BL/6 versus BALB/c female mice**. Thioglycollate-elicited peritoneal macrophages were prepared from 8-week-old C57BL/6 and BALB/c (5 of each) female mice as described previously [6]. The macrophages of each mouse were adhered to the bottom of 12 well plates (1 × 10^7 ^cells/well in 1 mL media) for 4 h, the cells were washed with media three times. The cells were cultured overnight in the fresh media (500 μL) after the final wash. The cells were treated with 100 μL dissolved in 0.2% DMSO of dexamethasone, 10 μM; mevinolin, 20 μM; δ-tocotrienol, 10 μM; α,-tocopherol, 100 μM; riboflavin, 40 μM; or quercetin-HCL, 40 μM for I h, followed by stimulation with LPS (10 *n*g/mL) of each treatment. The assay mixtures were incubated at room temperature for 4 h, and the assay mixtures were centrifuged at 2,000 rpm for 20 min. The cells were then harvested, and the total cellular RNA was extracted from each pellet with RNeasy mini kit according to manufacturer's instructions [7,17]. The total secretion of TNF-α, of each inhibitor was estimated in the supernatants by radio-immunoassay (ELISA) kit according to the manufacture directions [7,17]. The cells viability were very good (> 95%) in all the treatments [4,7]. Data are means ± SD, *n *= 5 per treatment, and triplicate analyses of each sample. The treatments 1-7 correspond to: 1. Control (macrophages + LPS + 0.2% DMSO); 2. dexamethasone; 3. mevinolin; 4. α,-tocopherol; 5. δ-tocotrienol; 6. riboflavin; 7. Quercetin-HCL. Values in a column with a different superscript symbols are significantly different at ***P ***< 0.05, A = values, B = Percentages.

**Figure 8:**
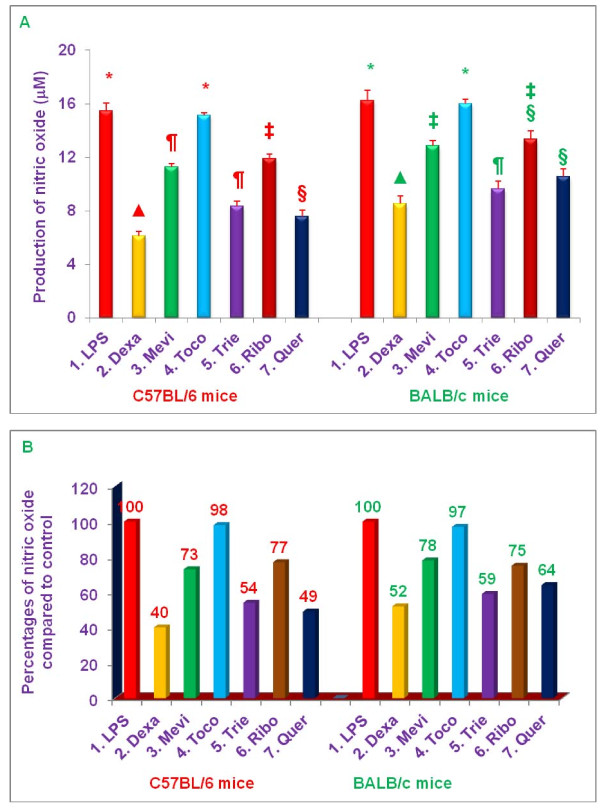
**Effects of various compounds on the productions of nitric oxide (NO) in LPS-stimulated (pre-treatment) peritoneal macrophages of 8-week-old C57BL/6 versus BALB/c female mice**. Thioglycollate-elicited peritoneal macrophages were prepared from 8-week-old C57BL/6 and BALB/c (5 of each) female mice as described previously [6]. The macrophages (1 x10^7 ^cells/well in 1.0 mL media) were adhered to the bottom of 100 mm tissue culture plate for 4 h. After 4 h the cells were treated with each compound (100 μL dissolved in 0.2% DMSO) of dexamethasone, 10 μM; mevinolin, 20 μM; α,-tocopherol, 100 μM; δ-tocotrienol, 10 μM; riboflavin, 40 μM; or quercetin-HCL, 40 μM for I h, followed by stimulation with LPS 10 *n*g/mL (10 μL of 1.0 μg/mL) of each treatment. The assay mixtures were incubated at room temperature for 4 h, after 4 h, the assay mixtures were centrifuged at 2,000 rpm for 20 min. The cells were then harvested, and the total cellular RNA was extracted from each pellet with RNeasy mini kit according to manufacturer's instructions [17]. The total level of NO for each inhibitor was estimated in the supernatant by using Griess reagent as described earlier [7,17]. The cells viability were very good (> 95%) in all the treatments [4,7]. Data are means ± SD, *n *= 5 per treatment, and triplicate analyses of each sample. The treatments 1-7 correspond to: 1. Control (macrophages + LPS + 0.2% DMSO); 2. dexamethasone; 3. mevinolin; 4. α,-tocopherol; 5. δ-tocotrienol; 6. riboflavin; 7. quercetin-HCL. Values in a column with a different superscript symbols are significantly different at ***P ***< 0.05, A = values, B = Percentages.

The anti-inflammatory properties of dexamethasone (10 μM), mevinolin (20 μM), δ-tocotrienol (10 μM), riboflavin (40 μM), quercetin (40 μM), and α,-tocopherol (100 μM) were further explored by pre-treating peritoneal macrophages for 1 h with each of these compounds followed by stimulation with LPS and measurement of TNF-α, (4 h) or nitrite (36 h) in culture supernatants, respectively, after LPS stimulation (Figures 7A, B and 8A, B).

All the inhibitors, except α,-tocopherol, inhibited LPS-induced secretion of TNF-α, by macrophages from C57BL/6 mice. Dexamethasone was the most potent inhibitor, yielded a 68% decrease in TNF-α, secretion (***P ***< 0.02), whereas, quercetin (37%), riboflavin (31%), mevinolin (24%), and δ-tocotrienol (20%) resulted in only moderate, but significant decreases (***P ***< 0.05), compared to controls (Figure 7A). Similarly, the results with macrophages derived from BALB/c mice paralleled those of macrophages from C57BL/6 mice; decreases in the levels of TNF-α, were observed with dexamethasone (72%; ***P ***< 0.02), quercetin (28%), riboflavin (25%), mevinolin (17%), and δ-tocotrienol (15%), compared to controls (Figure 7B). The extent to which TNF-α, secretion by LPS-stimulated macrophages was blocked by these various proteasome inhibitors was slightly greater for C57BL/6 mice than for BALB/c mice (~ 20%), though these differences were not statistically significant (Figure 7B).

Production of NO by LPS-stimulated macrophages from C57BL/6 and BALB/c mice was significantly (***P ***< 0.02) reduced by dexamethasone (60% and 48%, respectively), δ-tocotrienol (46% and 41%, respectively), and quercetin (51% and 36%, respectively), compared to respective controls (Figure 8A, B). However, mevinolin (27% and 22%) and riboflavin (23% and 25%) produced only moderate reductions in NO production compared to controls (Figure 8B). As with TNF-α, the extent to which NO production by LPS-stimulated macrophages was blocked by these various proteasome inhibitors was slightly greater for C57BL/6 mice than for BALB/c mice (~ 20%), though these differences were not statistically significant (Figure 8B). Thus, these studies clearly demonstrate that the proteasome inhibitors tested suppress secretion of TNF-α, and production of NO by LPS-stimulated thioglycolate-elicited peritoneal macrophages derived from C57BL/6 and BALB/c mice.

### Comparative effects of various proteasome inhibitors on TNF-α, secretion and NO production by LPS-stimulated thioglycolate-elicited peritoneal macrophages from C57BL/6, BALB/c, double knockout LMP7/MECL-1^-/-^, and PPAR-α,^-/- ^knockout mice

The next series of experiments were conducted with macrophages from mice that have aberrant responses to LPS. It has previously been well documented that macrophages from PPAR-α^-/- ^mice produce an unusually robust and inflammatory response to LPS [14,15]. Similarly, we have been studying the effect of role of various proteasome subunit knockouts on the inflammatory response to LPS [22,23]. Of particular relevance, we reported that LPS-stimulated peritoneal macrophages derived from double knockout LMP7/MECL-1^-/- ^mice generated a relatively normal TNF-α, response, but a markedly reduced NO response, compared to control (C57BL/6) mice [22]. Consequently, we thought it would be of value to determine the effect of proteasome inhibitors on TNF-α, and NO production by LPS-stimulated macrophages from these mouse strains. The experiments were carried out only with three lead, naturally-occurring non-toxic, commercially available compounds (δ-tocotrienol, riboflavin, and quercetin), which inhibit the secretion of TNF-α, and production of NO, which are increased during ageing process [2]. Therefore, the anti-inflammatory properties of δ-tocotrienol (10 μM), riboflavin (40 μM), quercetin (40 μM), and α,-tocopherol (100 μM; as a control)) were explored in LPS-induced thioglycolate-elicited peritoneal macrophages derived from knockout LMP7/MECL-1^-/- ^and PPAR-α,^-/- ^mice using identical conditions for this series of experiments as described in the previous paragraph for the measurement of TNF-α, (4 h) or nitrite (36 h) in culture supernatants, respectively, after LPS-stimulation with the expectation that these experiments would provide further insight into the mechanisms by which these proteasome inhibitors suppress inflammatory responses; macrophages from C57BL/6 and BALB/c mice were used as controls.

**Figure 9:**
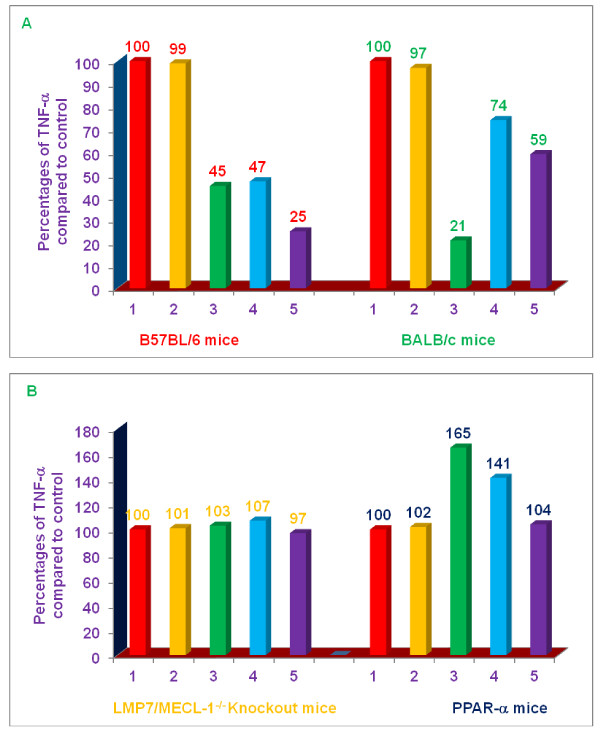
**Effects of various compounds on the secretion of TNF-α, in LPS-stimulated thioglycolate-elicited peritoneal macrophages of 8-week-old C57BL/6, BALB/c, double subunits knockout (LMP-7/MECL-1^-/-^), and PPAR-α,^-/- ^female mice**. Thioglycolate-elicited peritoneal macrophages of each mouse were adhered to the bottom of 12 well plates (1 × 10^7 ^cells/well in 1.0 mL media) for 4 h. After 4 h, the cells were washed with media three times. The cells were cultured overnight in the fresh media (500 μL) at 37°C in an incubator at 5% CO2 after the final wash. After 4 h the cells were treated with each compound (100 μL dissolved in 0.2% DMSO) of α,-tocopherol, 100 μM; δ-tocotrienol, 10 μM; riboflavin, 40 μM; or quercetin-HCL, 40 μM for I h, and the LPS (10 μL of 1.0 μg/mL) was added to assay mixture. The assay mixtures were incubated for 4 h at room temperature. The supernatants were collected after 4 h of LPS challenge, and stored at -70°C to carry out TNF-α, assay by ELISA. The cells viability were very good (> 95%) in all the treatments. The treatments 1-5 correspond to: 1. Control (media + macrophages (cells) + LPS + 0.2% DMSO); 2. α,-tocopherol; 3. δ-tocotrienol; 4. riboflavin; 5. quercetin-HCL.

**Figure 10:**
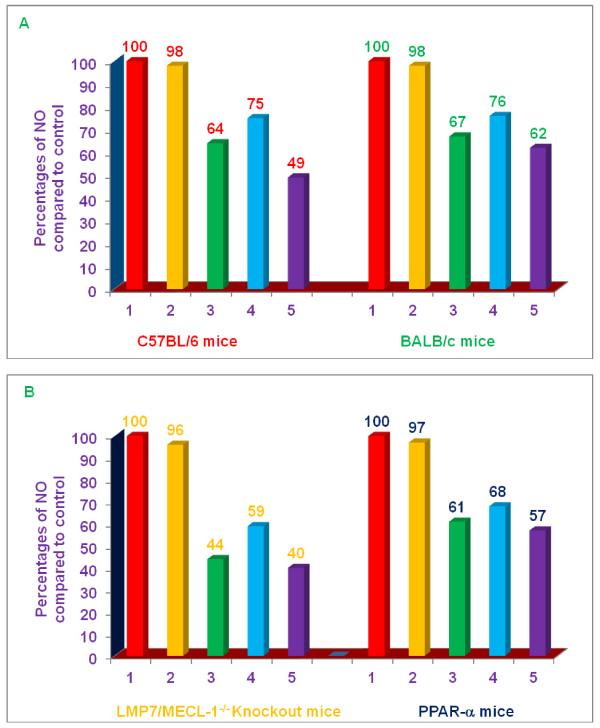
**Effects of various compounds on the induction of NO in LPS-stimulated thioglycolate-elicited peritoneal macrophages of 8-week-old C57BL/6, BALB/c, double subunits knockout (LMP-7/MECL-1-/-), and PPAR-α, female mice**. Thioglycolate-elicited peritoneal macrophages of each mouse were adhered to the bottom of 12 well plates (1 × 10^7 ^cells/well in 1.0 mL media) for 4 h. After 4 h, the cells were washed with media three times. The cells were cultured overnight in the fresh media (500 μL) at 37°C in an incubator at 5% CO2 after the final wash. The cells were treated with 100 μL dissolved in 0.2% DMSO of α,-tocopherol, 100 μM; δ-tocotrienol, 10 μM; riboflavin, 40 μM; or quercetin-HCL, 40 μM for I h, and the LPS + IFN-γ (10 μL of 1.0 μg/mL + 50 units/mL) was added to assay mixture. The assay mixtures were incubated for 36 h at room temperature. The supernatants were collected after 36 h of LPS challenge, and stored at -70°C to carry out NO estimation, using the Greiss reagent. The cells viability were very good (> 95%) in all the treatments. The treatments 1-5 correspond to: 1. Control (media + macrophages (cells) + LPS + IFN-γ + 0.2% DMSO); 2. α,-tocopherol; 3. δ-tocotrienol; 4. riboflavin; 5. quercetin-HCL.

LPS-induced TNF-α, secretion and NO production by macrophages derived from C57BL/6 and BALB/c mice were significantly inhibited (53% to 79% for TNF-α, and 24 to 51% for NO) by δ-tocotrienol, riboflavin, and quercetin; α,-tocopherol produced no inhibitory effect (Figure 9A). In contrast, none of these proteasome inhibitors suppressed secretion of TNF-α, in double knockout LMP7/MECL-1^-/- ^mice or PPAR-α,^-/- ^knockout mice compared to controls (Figure 9B). Interestingly, δ-tocotrienol and riboflavin actually produced significant increases in LPS-induced TNF-α, secretion by macrophages from PPAR-α,^-/- ^knockout mice. In marked contrast to our findings with TNF-α, all compounds, except α,-tocopherol significantly blocked the production of NO (32 to 60%) by LPS-stimulated macrophages from double knockout LMP7/MECL-1^-/- ^mice and PPAR-α,^-/- ^knockout mice, compared to controls, (Figure 10A, B).

We previously reported that NO production by LPS-stimulated peritoneal macrophages was markedly reduced in LMP7/MECL-1^-/- ^knockout mice; TNF-α, production, in contrast, was not markedly affected by the LMP7/MECL-1^-/- ^genotype [23]. We also demonstrated that in order to suppress TNF-α, production by LPS stimulated macrophages with proteasome inhibitors, both chymotrypsin- and trypsin-like proteasome activities must be suppressed [22]. Thus, the capacity of δ-tocotrienol, riboflavin, and quercetin to block TNF-α, production in C57BL/6 and BALB/c, but not in LMP7/MECL-1^-/-^, would be explained if these agents inhibited primarily the LMP2, LMP7 and MECL-1 subunits of mouse immunoproteasomes, with comparatively lower suppressive effects on X, Y, and Z subunits of constitutively expressed proteasomes. LPS-stimulation of macrophages from C57BL/6 and BALB/c would induce production of immunoproteasomes in which X, Y and Z subunits were partially replaced by LMP2, LMP7 and MECL-1^-/-^, decreased TNF-α, production would occur if these latter subunits were potently suppressed by δ-tocotrienol, riboflavin, and quercetin. In contrast, the × and Z components of LPS-stimulated macrophages from LMP7/MECL-1^-/- ^mice, could not be replaced by LMP7 and MECL-1^-/-^. If the chymotrypsin-like or trypsin-like activities of × and Z proteasome subunits were comparatively resistant to the inhibitory effects of δ-tocotrienol, riboflavin, and quercetin, TNF-α, production by LPS-stimulated macrophages from LMP7/MECL-1^-/- ^mice would be unaffected, since inhibition of both chymotrypsin- and trypsin-like proteasomal activities are required to suppress TNF-α, secretion.

The capacity of δ-tocotrienol, riboflavin, and quercetin to inhibit NO production by LPS-stimulated macrophages from LMP7/MECL-1^-/- ^mice might be explained by the high sensitivity of NO production to modulation of immunoproteasome subunits [22,23]. Thus, we previously demonstrated that LPS-stimulated macrophages from LMP2^-/- ^knockout mice produce substantially less NO than control littermates, and concluded from those experiments that inducible immunoproteasome subunits play a critical role in NO production [23]. Thus, NO production by LPS-stimulated macrophages from LMP7/MECL-1^-/- ^mice is very low, therefore IFN-γ has to be added concurrently to observe NO production and the defect is reversed. The present inhibitors suppress the LPS-induced NO production in the presence of IFN-γ (Figure 10A, B)

### Effect of various proteasome inhibitors on gene expression of TNF-α, and iNOS by LPS-stimulated thioglycolate-elicited peritoneal macrophages from C57BL/6, BALB/c, double knockout LMP7/MECL-1^-/-^, and PPAR-α,^-/- ^knockout mice

The experiments described above demonstrated that δ-tocotrienol, riboflavin, and quercetin inhibited secretion of TNF-α, by LPS-stimulated thioglycolate-elicited peritoneal macrophages from C57BL/6 and BALB/c, but not LMP7/MECL-1^-/- ^or PPAR-α,^-/- ^knockout mice (Figures 9A, B). In contrast, these compounds all inhibited NO production by LPS-stimulated thioglycolate-elicited peritoneal macrophages from all four strains of mice (Figure 10A, B). In order to determine whether these changes resulted from alterations in transcription of the relevant genes, we measured the effect of various proteasome inhibitors on mRNA levels for TNF-α, and iNOS in LPS-stimulated thioglycolate-elicited peritoneal macrophages from 8-week-old female C57BL/6, BALB/c, double knockout LMP7/MECL-1^-/-^, and PPAR-α,^-/- ^mice. The concentrations of each inhibitor and condition were similar to those used in earlier experiments. Cells were treated with δ-tocotrienol (10 μM), riboflavin (40 μM), and quercetin (40 μM) for 1 h, then LPS (10 μL of 1.0 μg/mL) was added to each well and incubated for an additional 4 h. Total cellular RNA was then extracted and reverse-transcribed, and gene analyses were carried out by RT-PCR and Southern blots analyses.

**Figure 11:**
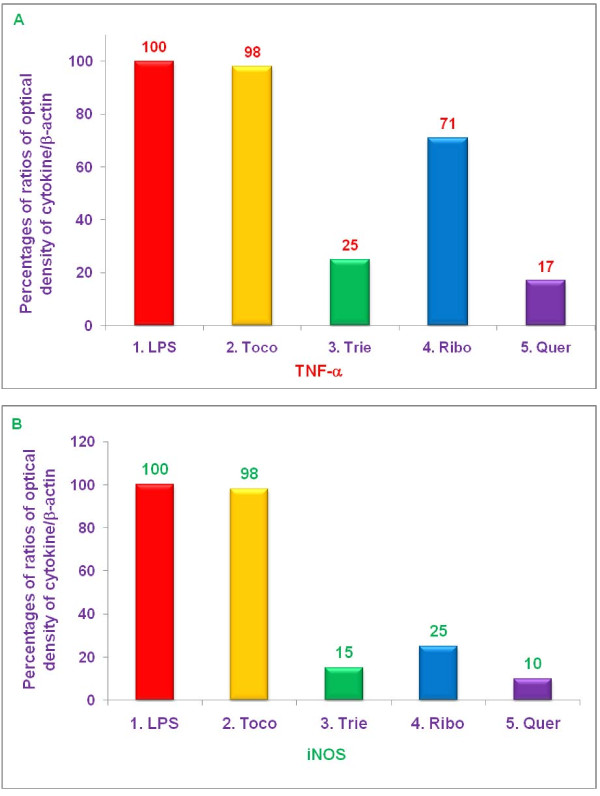
**The effect of proteasome inhibitors on gene expression of TNF-α, and iNOS in LPS-induced peritoneal macrophages from C57BL/6 mice**. Thioglycollate-elicited peritoneal macrophages were prepared from 8-week-old C57BL/6 mice, adhered to the bottom of 12 well plates (1 × 10^7 ^cells/well in 1 mL media) for 2 h, after 2 h, treated with 100 μL dissolved in 0.2% DMSO of α,-tocopherol (100 μM), δ-tocotrienol (10 μM), riboflavin (40 μM), or quercetin-HCL (40 μM) for 2 h. Then cells were challenged with LPS (10 *ng*/well; 400 μL), and incubated at room temperature for 4 h. Total cellular RNA was extracted and real-time polymerase chain reaction (RT-PCR) was conducted to quantitate expression of TNF-α, (A), and iNOS (B) genes. Data are presented as the percent of mRNA for each of the genes analyzed compared to controls. The treatments 1-5 correspond to: 1. Control (media + macrophages (cells) + LPS + 0.2% DMSO); 2. α,-tocopherol; 3. δ-tocotrienol; 4. riboflavin; 5. quercetin-HCL.

**Figure 12:**
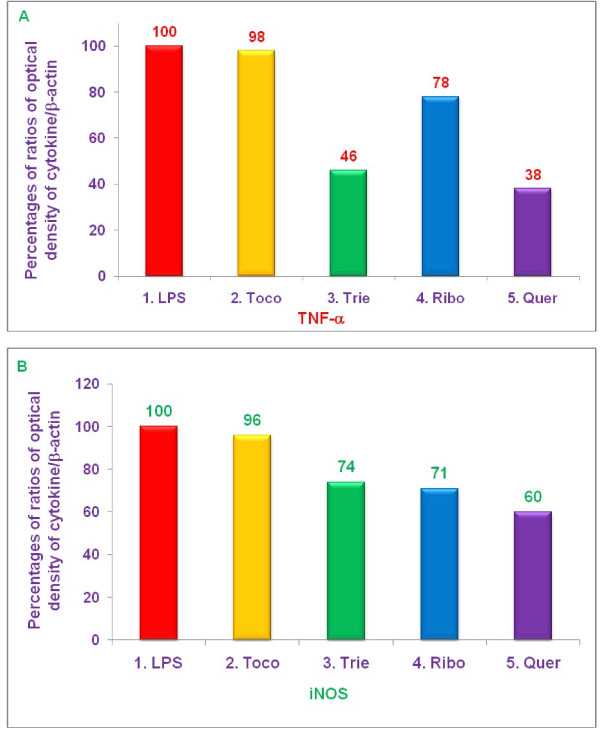
**The effect of proteasome inhibitors on gene expression of TNF-α, and iNOS in LPS-stimulated peritoneal macrophages from BALB/c mice**. Thioglycollate-elicited peritoneal macrophages were prepared from 8-week-old BALB/c mice, and adhered to the bottom of 12 wells plate (1 × 10^7 ^cells/well in 1 mL media) for 2 h, then treated with 100 μL dissolved in 0.2% DMSO of α,-tocopherol (100 μM), δ-tocotrienol (10 μM), riboflavin (40 μM), or quercetin-HCL (40 μM) for 2 h. After 2 h, cells were challenged with LPS (10 *ng*/well; 400 μL), and incubated at room temperature for 4 h. Total cellular RNA was extracted and real-time polymerase chain reaction (RT-PCR) was conducted to quantitate expression of TNF-α, (A), and iNOS (B) genes. Cell viability exceeded > 95%) in all the treatments. Data are presented as the percent of mRNA for each of the genes analyzed compared to controls. The treatments 1-5 correspond to: 1. Control (media + cells + LPS + 0.2% DMSO); 2. α,-tocopherol; 3. δ-tocotrienol; 4. riboflavin; 5. quercetin-HCL.

Each of the above proteasome inhibitors tested, except α,-tocopherol, significantly reduced mRNA expression for TNF-α, and iNOS in LPS-stimulated, thioglycolate-elicited peritoneal macrophages derived from female C57BL/6 mice (Figure 11A, B). Gene expression for TNF-α, and iNOS was markedly reduced by treatment with δ-tocotrienol (75% and 85%, respectively), riboflavin (29% and 75%, respectively), and quercetin (83% and 90%, respectively), compared to controls (Figure 11A, B). With LPS-stimulated macrophages from BALB/c mice, there was also significant reduction (***P ***< 0.05) of gene expression for TNF-α, and iNOS by δ-tocotrienol (54% and 26%, respectively), riboflavin (22% and 29%, respectively), and quercetin (62% and 40%, respectively), compared to controls, though the extent of inhibition was generally lower than that observed with C57BL/6 mice (Figure 12A, B).

**Figure 13:**
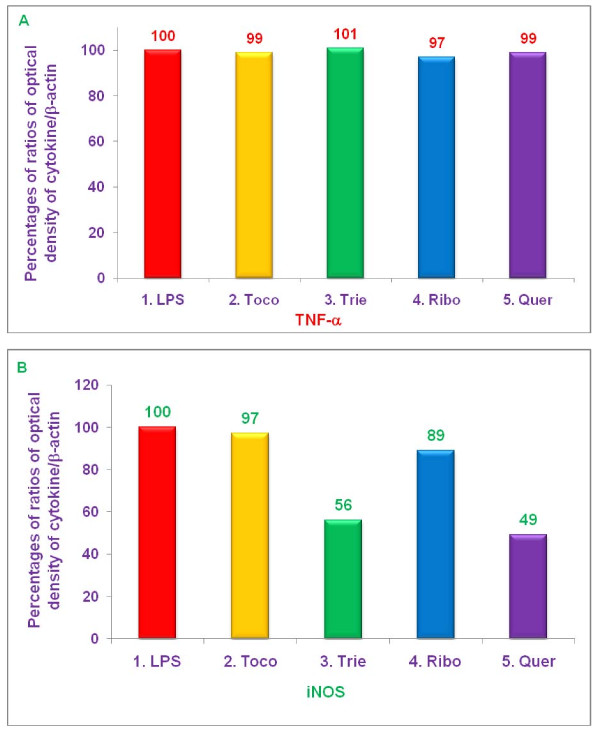
**The effect of proteasome inhibitors on gene expression of TNF-α, and iNOS in LPS-stimulated peritoneal macrophages from LMP7/MECL-1^-/- ^knockout mice**. Thioglycollate-elicited peritoneal macrophages were prepared from 8-week-old LMP7/MECL-1^-/- ^knockout mice, adhered to the bottom of 12 wells plate (1 × 10^7 ^cells/well in 1 mL media) for 2 h, then treated with 100 μL dissolved in 0.2% DMSO of α,-tocopherol (100 μM), δ-tocotrienol (10 μM), riboflavin (40 μM), or quercetin-HCL (40 μM) for 2 h. After 2 h, the cells were challenged with LPS + IFN-γ (10 *ng*/well; 400 μL + 50 units/mL), and incubated at room temperature for 4 h. Total cellular RNA was extracted and real-time polymerase chain reaction (RT-PCR) was conducted to quantitate expression of TNF-α, (A), and iNOS (B) genes. Cell viability exceeded > 95%) in all the treatments. Data are presented as the percent of mRNA for each of the genes analyzed compared to controls. The treatments 1-5 correspond to: The treatments 1-5 correspond to: 1. Control (media + cells + LPS + 0.2% DMSO); 2. α,-tocopherol; 3. δ-tocotrienol; 4. riboflavin; 5. quercetin-HCL.

In LPS-stimulated macrophages from LMP7/MECL-1^-/- ^knockout mice, the pattern of inhibition of mRNA production for TNF-α, and iNOS genes by proteasome inhibitors varied markedly from that observed with either C57BL/6 or BALB/c mice. None of the proteasome inhibitors had any effect on TNF-α, mRNA expression (Figure 13A), but inhibition of iNOS mRNA was attained with δ-tocotrienol (44%), riboflavin, (11%) and quercetin (51%) compared to control (Figure 13B).

**Figure 14:**
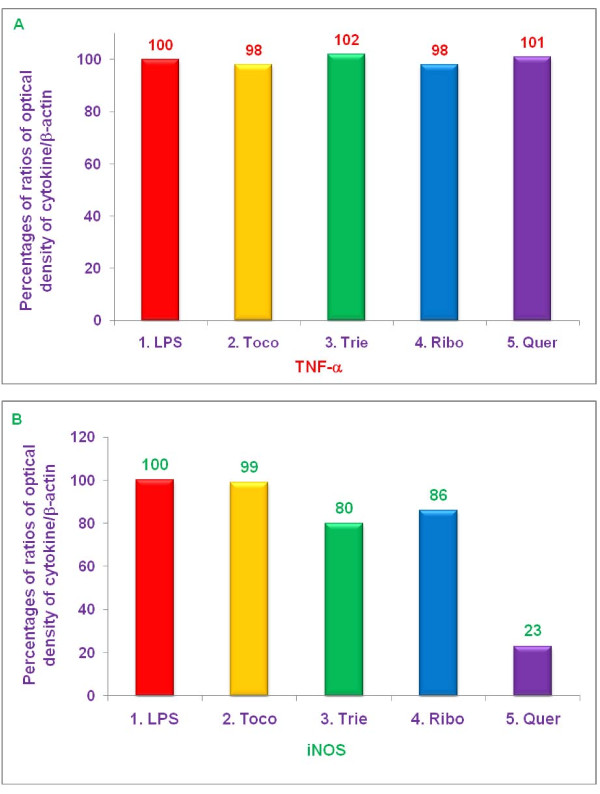
**The effect of proteasome inhibitors on gene expression of TNF-α, and iNOS in LPS-stimulated peritoneal macrophages from PPAR-α,^-/- ^knockout mice**. Thioglycollate-elicited peritoneal macrophages were prepared from 8-week-old PPAR-α,^-/- ^knockout mice, adhered to the bottom of 12 wells plate (1 × 10^7 ^cells/well in 1 mL media) for 2 h, and treated with 100 μL dissolved in 0.2% DMSO of α,-tocopherol (100 μM), δ-tocotrienol (10 μM), riboflavin (40 μM), or quercetin-HCL (40 μM) for 2 h. Then cells were challenged with LPS (10 *ng*/well; 400 μL), and incubated at room temperature for 4 h. Total cellular RNA was extracted and real-time polymerase chain reaction (RT-PCR) was conducted to quantitate expression of TNF-α, (A), and iNOS (B) genes. Cell viability exceeded > 95%) in all the treatments. Data are presented as the percent of mRNA for each of the genes analyzed compared to controls. The treatments 1-5 correspond to: The treatments 1-5 correspond to: 1. Control (media + cells + LPS + 0.2% DMSO); 2. α,-tocopherol; 3. δ-tocotrienol; 4. riboflavin; 5. quercetin-HCL.

In LPS-stimulated macrophages from PPAR-α,^-/- ^knockout mice, the pattern of inhibition of mRNA production for TNF-α, and iNOS genes by proteasome inhibitors also varied markedly from that observed with either C57BL/6 or BALB/c mice, and more closely resembled the pattern obtained with LMP7/MECL-1^-/- ^knockout mice. As with LMP7/MECL-1^-/- ^mice, proteasome inhibitors did not affect TNF-α, mRNA levels (Figure 14A). Treatment with quercetin markedly suppressed iNOS mRNA levels (77%); iNOS gene expression was inhibited less profoundly by δ-tocotrienol (20%) and riboflavin (14%) (Figure 14B). In summary, results of mRNA expression studies were generally consistent with those of TNF-α, secretion and NO production, though the enhanced secretion of TNF-α, by LPS-stimulated macrophages from PPAR-α,^-/- ^knockout mice treated with riboflavin and δ-tocotrienol was not explained by a corresponding increase in mRNA levels. Moreover, quercetin was found to be slightly better anti-inflammatory compound for the above mentioned markers than δ-tocotrienol and riboflavin.

## Discussion

The primary objectives in the present study were to further evaluate several naturally-occurring proteasome inhibitors for their capacity to suppress inflammatory processes, and to define mechanisms responsible for these anti-inflammatory effects. Anti-inflammatory properties of the proteasome inhibitors were evaluated in macrophages derived from several sources (e.g. the RAW 264.7 cell line, and thioglycolate-elicited peritoneal macrophages from four strains of mice (C57BL/6, BALB/c, double knockout LMP7/MECL-1^-/- ^and PPAR-α,^-/-^). As a result of these studies we have identified several naturally-occurring proteasome inhibitors that could potentially decrease levels of inflammatory cytokines and NO that may contribute to the development of diseases associated with ageing.

First, we demonstrated that dexamethasone, mevinolin, δ-tocotrienol, riboflavin and quercetin are potent inhibitors of chymotrypsin-like activity of 20S rabbit muscle proteasomes, and that (-) Corey lactone and amiloride enhanced this activity. We also demonstrated that dexamethasone, mevinolin, δ-tocotrienol, riboflavin and quercetin inhibited the secretion of TNF-α and NO production by LPS-stimulated RAW 264.7 macrophage like murine cell cultures. Further, levels of NF-κwithin the nucleus were decreased, whereas cellular P-Iκlevels were increased, by pre-treatment of LPS-stimulated RAW 264.7 cells with dexamethasone, mevinolin, δ-tocotrienol, riboflavin and quercetin.

NF-κis maintained in an inactive state in the cytoplasm of cells when it is bound to Iκ. LPS induces a series of events that results in phosphorylation and ubiquitination of Iκwith subsequent degradation by the proteasome. These actions result in NF-κactivation, translocation to the nucleus, and increased transcription of several genes encoding pro-inflammatory cytokines. Consequently, the capacity of dexamethasone, mevinolin, δ-tocotrienol, riboflavin and quercetin to inhibit proteasome activity in conjunction with their capacity to increase cellular levels of P-Iκand decrease nuclear translocation of NF-κ, suggests that the mechanism by which these agents suppress production of TNF-α, and NO involves decreased degradation of ubiquinated P-Iκby the proteasome, resulting in depressed translocation of NF-κto the nucleus. Therefore, ultimately, these proteasome inhibitors suppress production of TNF-α, and NO, and exert their anti-inflammatory effects by inhibiting NF-κB, activation.

This conclusion was further supported by experimental testing of these inhibitors (dexamethasone, mevinolin, δ-tocotrienol, riboflavin and quercetin) in LPS-stimulated thioglycolate-elicited peritoneal macrophages derived from four different strains of mice. All inhibitors significantly inhibited LPS-induced secretion of TNF-α by macrophages derived from C57BL/6 and BALB/c mice (Figure 9A). Although, macrophages derived from C57BL/6 mice compared to BALB/c mice yielded slightly better (20%) inhibition in LPS-stimulated secretion of TNF-α (Figure 9A). In marked contrast to the results attained with C57BL/6 and BALB/c mice, TNF-α, secretion was essentially unaffected by treatment of LPS-stimulated macrophages derived from LMP7/MECL-1^-/- ^knockout mice with δ-tocotrienol, riboflavin, and quercetin (9B). Similarly, these compounds failed to suppress TNF-α, secretion by LPS-stimulated macrophages derived from PPAR-α,^-/- ^knockout mice (since activation of PPAR-α^-/- ^mice normally reduced inflammation); δ-tocotrienol and riboflavin treatment actually enhanced TNF-α, secretion (Figure 9B). In marked contrast to the observations with TNF-α, δ-tocotrienol, riboflavin, and quercetin suppressed NO production by LPS-stimulated macrophages from C57BL/6, BALB/c, LMP7/MECL-1^-/-^, and PPAR-α,^-/- ^knockout mice (Figure 10A, B). The effects of these proteasome inhibitors on mRNA production by LPS-stimulated macrophages were generally consistent with at the protein levels of TNF-α, secretion and NO production.

Our previously published studies strongly support the concept that proteasomes are key regulators of LPS-stimulated inflammatory signaling pathways (3,4,6-8). Proteolytic activity of proteasomes is mediated by the 20S proteasomes, a hollow, cylindrical multi-protein complex consisting of three proteolytic subunits, X, Y, and Z, with chymotrypsin-like, trypsin-like, and post-glutamase activities, respectively. A variety of inflammatory stimuli induce alterations in newly assembled "immuno-proteasomes" in which X, Y and Z subunits are partially replaced by LMP7, LMP2, and MECL-1, respectively.

We previously demonstrated that low doses of lactacystin, which suppresses primarily chymotrypsin-like activity of the proteasome, potently suppressed production of NO, but not TNF-α, by LPS stimulated macrophages. We further demonstrated that in order to suppress TNF-α, secretion by LPS-stimulated macrophages with proteasome inhibitors, both chymotrypsin-like and trypsin-like proteasome activities must be suppressed (22). Subsequent experiments with proteasome subunit knockout (LMP7^-/-^, LMP2^-/-^, MECL-1^-/-^, and LMP7/MECL-1^-/-^) revealed that NO production by LPS-stimulated peritoneal macrophages was markedly reduced in LMP7^-/-^, LMP2^-/-^, MECL-1^-/-^, and LMP7/MECL-1^-/-^knockout mice; TNF-α, production, in contrast, was not markedly affected by any of these knockout genotypes [23].

In the current study, the capacity of the investigated proteasome inhibitors to inhibit TNF-α, secretion by LPS-stimulated macrophages from several sources (i.e. the RAW 264.7 cell line, and peritoneal macrophages from C57BL/6 and BALB/c mice) supports the conclusion that these inhibitors suppress both chymotrypsin-like and trypsin-like activities of the proteasomes, since both of these activities must be suppressed in order to inhibit TNF-α, secretion. These conclusions were supported by our analysis of the effects of these inhibitors on proteolytic activity of the proteasome.

The capacity of δ-tocotrienol, riboflavin, and quercetin to block TNF-α, secretion in C57BL/6 and BALB/c, but not in LMP7/MECL-1^-/- ^mice, is an intriguing result. LPS-stimulation of macrophages from C57BL/6 and BALB/c mice would be expected to induce production of immunoproteasomes in which X, Y and Z subunits were partially replaced by LMP2, LMP7 and MECL-1^-/-^. The × and Z components of LPS-stimulated macrophages from LMP7/MECL-1^-/- ^mice, however, cannot be replaced by LMP7 and MECL-1^-/-^. The macrophages from knockout mice when induced produce robust amounts of TNF-α,. Thus, the differential capacity of δ-tocotrienol, riboflavin, and quercetin to inhibit TNF-α, secretion by LPS-stimulated macrophages from C57BL/6 and BALB/c vs LMP7/MECL-1^-/- ^knockout mice would appear to be attributable to a differential susceptibility of × and Z vs LMP7 and MECL-1 proteasomal subunits to inhibition by these inhibitors.

Presumably δ-tocotrienol, riboflavin, and quercetin are potent inhibitors of LMP2, LMP7 and MECL-1^-/- ^subunits of mouse immunoproteasomes, with comparatively lesser suppressive effects on X, Y, and Z subunits of constitutively expressed proteasomes. Thus, the chymotrypsin-like and trypsin-like activities of normal immunoproteasomes (containing LMP2, LMP7 and MECL-1^-/-^) from C57BL/6 and BALB/c mice would be suppressed by δ-tocotrienol, and quercetin, resulting in decreased TNF-α, secretion. In contrast, the chymotrypsin-like and trypsin-like activity of proteasomes (containing X, Y, Z, and LMP2) from LMP7/MECL-1^-/- ^knockout mice would not be suppressed by δ-tocotrienol, riboflavin, and quercetin, resulting in normal TNF-α, secretion.

The finding that δ-tocotrienol, riboflavin, and quercetin inhibited NO production by LPS-stimulated macrophages from C57BL/6, BALB/c, LMP7/MECL-1^-/-^, and PPAR-α,^-/- ^mice was also quite interesting; the LMP7/MECL-1^-/- ^genotype mice did not affect susceptibility to inhibition by these compounds. These macrophages from knockout mice do not induce very much NO, therefore IFN-γ had to be added along with LPS to induce NO in cells that have X, Y, Z, and LMP2 subunits [22].

PPAR-α,^-/- ^knockout mice have exaggerated inflammatory responses to a variety of stimuli, because activation of PPAR-α, leads to anti-inflammatory effects [24]. The mechanisms leading to these exaggerated inflammatory responses are not clearly understood, but are believed to be at least partially attributable to increased NFκB activity [24,25]. Consequently, one would expect TNF-α, secretion by LPS-stimulated macrophages from PPAR-α,^-/- ^knockout mice to be highly up-regulated with respect to TNF-α, secretion and relatively resistant to inhibition by proteasome inhibitors that degrade IκB, and decrease NFκB activity. Therefore, we found that δ-tocotrienol, riboflavin, and quercetin failed to suppress TNF-α, secretion by LPS-stimulated macrophages from PPAR-α,^-/- ^knockout mice. In fact TNF-α, secretion was substantially enhanced by riboflavin and δ-tocotrienol. A clearer explanation of these results will be dependent on further elucidation of interactions between PPAR-α, and ubiquitin proteasome pathways [26].

Our present results demonstrate that δ-tocotrienol, riboflavin, and quercetin are naturally-occurring potent proteasome inhibitors for the inhibition of NO production tested *in vitro*. These results also confirms earlier report that γ-tocotrienol blocked LPS-stimulated activation of NF-κB, and also blocked TNF-α, induced phosphorylation and degradation of IκBα, through the inhibition of IκBα, kinase activation [27]. Tocotrienols have been shown to modestly inhibit or activate the proteasomal activity depending on its concentrations (Tables 1, [9]). Therefore, blocking the proteasomal activity with low doses of tocotrienols could potentially reduce inflammatory responses, but at high doses of tocotrienols may cause apoptotic cell death in cancer [9,28]. Quercetin, on the other hand is a natural proteasome inhibitor that can affect several proteasomal activities.

## Conclusions

The present study describes the possible mechanism of inhibition of NO production (released during ageing processes or acute and chronic disease) by naturally-occurring proteasome inhibitors (quercetin, δ-tocotrienol, and riboflavin). Dexamethasone, mevinolin, δ-tocotrienol, riboflavin and quercetin were all found to be potent inhibitors of chymotrypsin-like activity of 20S rabbit muscle proteasomes. δ-Tocotrienol, riboflavin, and quercetin also inhibited chymotrypsin-like, trypsin-like and post-glutamase activities of the proteasomes in RAW 264.7 cells. These compounds also blocked LPS-stimulated secretion of TNF-α, NO production, activation of NF-κB, and degradation of P-IκB in RAW 264.7 murine macrophages. Similarly, these compounds suppressed TNF-α, secretion and NO production by LPS-stimulated peritoneal macrophages derived from C57BL/6 and BALB/c mice. δ-Tocotrienol, riboflavin, and quercetin blocked the LPS-stimulated production of NO and had no effect on the secretion of TNF-α, in macrophages derived from LMP7/MECL-1^-/- ^and PPAR-α,^-/- ^knockout mice. These results indicate that δ-tocotrienol, riboflavin, and quercetin treatments differentially inhibit the secretion of TNF-α, of LPS-stimulated macrophages derived from C57BL/6, BALB/c versus LMP7/MECL-1^-/- ^and PPAR-α,^-/- ^knockout mice. Moreover, all the gene expression results of TNF-α, and iNOS genes are generally consistent with the results of protein levels of secretion of TNF-α, and production of NO observed with either C57BL/6, BALB/c, LMP7/MECL-1^-/-^, or PPAR-α,^-/- ^mice. All these results indicate that the production of NO may be modulated via NF-κB pathway, and δ-tocotrienol acts as a proteasome inhibitor at lower doses and as activator at higher doses (Table 1). These collective properties of δ-tocotrienol, riboflavin, and quercetin can be referred to as **"novel proteasome inhibitors"**, which might be used to suppress the production of inflammatory mediators in ageing humans, thereby decreasing the risk of developing a variety of age-related diseases that appear to be at least partially attributable to aberrant control of inflammatory responses with increasing age.

## Abbreviations

LPS: lipopolysaccharide; TNF-α: tumor necrosis factor-α; IL-1β: interleukin-1β; IL-6: interleukin-6; NO: nitric oxide; iNOS: nitric oxide synthase; NF-κB: nuclear factor-kappaB; P-IκB: phosphorylated-inhibitorkappaB; PPAR-α^-/-^: peroxisome proliferator-activated receptor-α^-/-^; 1. LPS (control group): media + cells + LPS (10 *ng*/mL) + 0.2%-0.4% dimethyl sulfoxide; 2. Dexa: dexamethasone; 3. Mev: mevinolin; 4. Toco: α,-tocopherol; 5. Trie: δ-tocotrienol; 6. Ribo: riboflavin; 7. Quer: quercetin.

## Authors' information

^a^Department of Basic Medical Sciences, University of Missouri-Kansas City, 2411 Holmes Street, Kansas City, MO 64108, USA.

^b^Department of Medicine, University of Kansas Medical Center, 3901 Rainbow Boulevard, Kansas City, KS 66160, USA.

^c^Division of Pharmacology and Toxicology, School of Pharmacy, University of Missouri-Kansas City, 2464 Charlotte Street, Kansas City, MO 64108, USA.

## Competing interests

The authors declare that they have no competing interests.

## Authors' contributions

All the authors were involved in the designing of the study. Dr. XT (Postdoctoral fellow M.S.) carried out TNF-α, NO, NF-κB and gene expression assays. Ms. JCR has carried out assays of 20S proteasomes and P-IκB assays. Dr. MZB has provided and kept the colony of PPAR-α,^-/- ^mice and has supervised Ms. JCR for carrying out previously mentioned assays. Dr. CJP edited the manuscript. All the authors have read and approved the final version.
